# Population pharmacokinetic and exposure–response analyses of elotuzumab plus pomalidomide and dexamethasone for relapsed and refractory multiple myeloma

**DOI:** 10.1007/s00280-021-04365-4

**Published:** 2021-11-26

**Authors:** Takafumi Ide, Mayu Osawa, Kinjal Sanghavi, Heather E. Vezina

**Affiliations:** Clinical Pharmacology and Pharmacometrics, Bristol-Myers Squibb, Route 206 and Province Line Road, Princeton, NJ 08648 USA

**Keywords:** Elotuzumab, Multiple myeloma, Pharmacokinetics, Progression-free survival, Safety

## Abstract

**Purpose:**

Elotuzumab plus pomalidomide/dexamethasone (E-Pd) demonstrated efficacy and safety in relapsed and refractory multiple myeloma (RRMM). The clinical pharmacology of elotuzumab [± lenalidomide/dexamethasone (Ld)] was characterized previously. These analyses describe elotuzumab population pharmacokinetics (PPK), the effect of Pd, and assess elotuzumab exposure–response relationships for efficacy and safety in patients with RRMM.

**Methods:**

A previously established PPK model was updated with E-Pd data from the phase 2 ELOQUENT-3 study (NCT02654132). The dataset included 8180 serum concentrations from 440 patients with RRMM from 5 clinical trials. Elotuzumab PK parameter estimates were used to generate individual daily time-varying average concentrations (daily C_avg_) for multi-variable time-to-event exposure–response analyses of progression-free survival (PFS) and time to the first occurrence of grade 3 + adverse events (AEs) in RRMM.

**Results:**

Elotuzumab PK were well-described by a two-compartment model with parallel linear and Michaelis–Menten elimination from the central compartment (V_max_) and non-renewable target-mediated elimination from the peripheral compartment (K_int_). Co-administration with Pd resulted in a 19% and 51% decrease in elotuzumab linear clearance and K_int_, respectively, versus Ld; steady-state exposures were similar. V_max_ increased with increasing serum M-protein. Hazard ratios (95% confidence intervals) for daily C_avg_ were 0.9983 (0.9969–0.9997) and 0.9981 (0.9964–0.9998) for PFS and grade 3 + AEs, respectively.

**Conclusions:**

The PPK model adequately described the data and was appropriate for determining exposures for exposure–response analyses. There were no clinically relevant differences in elotuzumab exposures between Pd and Ld backbones. In ELOQUENT-3, increasing elotuzumab daily C_avg_ prolonged PFS without increasing grade 3 + AEs.

**Supplementary Information:**

The online version contains supplementary material available at 10.1007/s00280-021-04365-4.

## Introduction

Multiple myeloma (MM) is the second most prevalent blood cancer, representing ~ 10% of hematologic malignancies [1–3]. Proteasome inhibitors (PIs), immunomodulatory drugs (IMiDs), and monoclonal antibodies are key treatment options for patients with MM [4]. Many patients now receive an IMiD (lenalidomide) and a PI (bortezomib) as part of their first lines of therapy. As such, patients with early-stage MM refractory to lenalidomide and bortezomib reflect a growing population. Typical treatment approaches for patients with relapsed and refractory multiple myeloma (RRMM) include within-class or between-class switches or the addition of an agent with a new mechanism of action. Pomalidomide, a third-generation IMiD that exerts potent, direct tumoricidal and immune-stimulating effects, is approved in the United States in combination with dexamethasone for patients who have received at least two prior therapies including lenalidomide and a PI and have experienced disease progression on or within 60 days of completion of the last therapy [5, 6]. The triplet regimens of elotuzumab, daratumumab, or isatuximab-irfc, each in combination with pomalidomide and dexamethasone, are also approved in the United States for patients who have received at least two prior therapies, including an IMiD and a PI [7–9]. In addition, the National Comprehensive Cancer Network (NCCN) guidelines recommend the use of bortezomib or ixazomib in combination with pomalidomide and dexamethasone as a preferred option for patients who have been previously treated for MM [10]. NCCN guidelines also endorse the use of either carfilzomib or cyclophosphamide together with pomalidomide and dexamethasone in this population [10].

Elotuzumab is a humanized immunoglobulin G1 immunostimulatory monoclonal antibody that targets signaling lymphocytic activation molecule family member 7 (SLAMF7) [11]. SLAMF7 is a cellular glycoprotein that is highly expressed on MM cells, natural killer (NK) cells, and some immune cells, but has a minimal expression on normal tissues [11, 12]. Elotuzumab has multiple mechanisms of action against MM cells, including NK cell–mediated antibody-dependent cellular cytotoxicity, directly activating NK cells, and macrophage-mediated NK cell killing of MM cells [11–16]. Elotuzumab is hypothesized to have synergistic effects with pomalidomide on NK cells, which could translate into the improved outcomes observed in patients with RRMM [17].

In phase 2 randomized ELOQUENT-3 study (NCT02654132) in patients with RRMM who received at least 2 prior therapies including lenalidomide and a PI, the addition of elotuzumab to pomalidomide and dexamethasone (E-Pd) reduced the risk of progression or death by 46% versus pomalidomide and dexamethasone alone [Pd; hazard ratio (HR) 0.54, 95% confidence interval (CI) 0.34–0.86, *p* = 0.008] [17]. In addition, the median progression-free survival (PFS) was 10.3 months in the E-Pd group and only 4.7 months in the Pd group. In ELOQUENT-3, patients received elotuzumab 10 mg/kg intravenously (IV) once every week (QW) during Cycles 1 and 2 (28 day cycles) followed by 20 mg/kg IV every four weeks (Q4W) thereafter. This early switch to a Q4W dosing regimen was selected using modeling and simulation with the goal of improving patient and provider convenience while maintaining the benefit–risk profile of elotuzumab in combination therapy.

The clinical pharmacology of elotuzumab as a monotherapy or in combination with lenalidomide and dexamethasone (E-Ld) has been characterized previously [18]. Elotuzumab exhibited nonlinear pharmacokinetics with target-mediated drug disposition. Efficacious concentrations of elotuzumab were achieved rapidly in the first two 28 day cycles following 10 mg/kg IV QW dosing and were maintained after switching to 10 mg/kg IV every 2 weeks (Q2W) dosing beginning at Cycle 3, which is the approved regimen in combination with lenalidomide and dexamethasone (Ld). Patients with higher baseline serum M-protein concentrations also appeared to have a faster target-mediated elimination of elotuzumab [18].

In the present work, using data from ELOQUENT-3, a previously established elotuzumab population pharmacokinetic model was updated to evaluate the effect of concomitant Pd on non-specific clearance and time-varying serum M-protein on target-mediated elimination. The objectives of the study were to characterize the pharmacokinetics of elotuzumab and the effect of covariates of interest on the pharmacokinetic parameters, as well as to describe the relationship between elotuzumab exposures and efficacy (measured by PFS), and elotuzumab exposures and safety [measured by time to the first occurrence of grade 3 + adverse events (AEs)] in patients with RRMM. Exposure comparisons were also made between elotuzumab 10 mg/kg IV Q2W and 20 mg/kg IV Q4W maintenance dosing regimens.

## Materials and methods

### Patients

The patients included in the population pharmacokinetic analysis had participated in one of five completed clinical studies that evaluated elotuzumab as monotherapy for high-risk smoldering myeloma [CA204-011 (NCT01441973)], in combination with Ld for RRMM [CA204-004 or ELOQUENT-2 (NCT01239797), CA204-005 (NCT01241292), CA204-007 (NCT01393964)], or in combination with Pd for RRMM [CA204-125 (NCT02654132) or ELOQUENT-3]. CA204-011 was a phase 2 study to assess the association between NK cell status and efficacy of elotuzumab monotherapy (10 mg/kg or 20 mg/kg) using biomarkers [19]. CA204-005 and CA204-007 were phase 1 studies of elotuzumab (10 mg/kg IV QW for two 28 day cycles followed by 10 mg/kg IV Q2W) plus Ld in Japanese patients with RRMM, and in patients with MM and varying degrees of renal function, respectively [20, 21]. ELOQUENT-2 was the phase 3 registrational study of elotuzumab (10 mg/kg IV QW for two 28 day cycles followed by 10 mg/kg IV Q2W) plus Ld versus Ld alone in patients with RRMM [22]. Finally, ELOQUENT-3 evaluated elotuzumab (10 mg/kg IV QW for two 28 day cycles followed by 20 mg/kg IV Q4W) plus Pd versus Pd alone in patients with RRMM [23]. Only the patients from ELOQUENT-3 were included in the exposure–response analyses of efficacy and safety. Baseline patient demographics and laboratory values for the population pharmacokinetic and exposure–response analyses are summarized in Table 1 and Supplementary Table S1, respectively. All studies were conducted in accordance with the principles of the Declaration of Helsinki and the International Conference on Harmonisation Guidelines for Good Clinical Practice. The Institutional Review Board committees at the various study centers approved each study protocol. All patients provided written informed consent.

**Table 1:** Summary statistics of baseline patient characteristics in the population pharmacokinetics dataset (*N* = 440)

Covariate	*N* (%)	Mean (SD)	Median (min, max)
Age (years)		65.5 (9.8)	66 (37, 88)
Bodyweight (kg)		75.6 (16.7)	75 (40, 150)
Sex			
Male	258 (59)		
Female	182 (41)		
Race			
White	351 (80)		
Black/African American	24 (6)		
Asian	55 (13)		
Other/Pacific Islander	10 (2)		
eGFR (mL/min/1.73 m^2^)		74.1 (23.5)	77.6 (4.58, 124)
M-protein (g/dL)		2.25 (1.58)	2.05 (0, 7.7)
Beta-2 microglobulin (mg/dL)		0.43 (0.371)	0.32 (0.04, 3.47)
Lactate dehydrogenase (U/L)		248 (153)	199 (54, 1900)
Albumin (g/dL)		3.81 (0.565)	3.8 (1.9, 5.0)
Renal function			
Normal	125 (28)		
Mild impairment	200 (46)		
Moderate impairment	92 (21)		
Severe impairment	13 (3)		
Renal failure	9 (2)		
Missing	1 (< 1)		
Hepatic function			
Normal	400 (91)		
Mild impairment	38 (9)		
Moderate impairment	1 (< 1)		
Missing	1 (< 1)		
ECOG performance status			
0	222 (51)		
1	188 (43)		
2	30 (7)		
Co-administration			
Monotherapy	31 (7)		
Lenalidomide/dexamethasone	349 (79)		
Pomalidomide/dexamethasone	60 (14)		
Anti-drug antibodies			
Never detected	336 (76)		
Detected at least once	102 (23)		
All observations are missing	2 (< 1)		

*N* (%) of patients with missing data: M-protein, 8 (2%); beta-2 microglobulin, 10 (2%); lactate dehydrogenase, 30 (7%)

*ECOG* Eastern Cooperative Oncology Group, *eGFR* estimated glomerular filtration rate; SD, standard deviation

### Quantification of elotuzumab in human serum

Elotuzumab serum concentrations were measured using a validated quantitative enzyme-linked immunosorbent assay with a lower limit of quantification of 190 ng/mL. Method TLIAM-0180 (Tandem Labs, West Trenton, NJ) was used for studies CA204004, CA204005, CA204007, and CA204011, and method BAL-II/MOA/019 (Syngene International Limited, Bangalore, India) was used for study CA204125. Cross validation was performed between these two methods using quality controls and pooled incurred samples, which met the cross-validation criteria. Accuracy, expressed as percentage deviation, ranged from 2.6% to 12.6% for method TLIAM-80, and − 6.27% to 3.27% for method BAL-II/MOA/019. Within and between assay variability expressed as coefficient of variation (%) ranged from 6.3% to 16.1% and 3.7% to 10.9%, respectively, for method TLIAM-80, and 1.48% to 18.10% and 3.22% to 8.89%, respectively, for method BAL-II/MOA/019.

### Analysis datasets

A population pharmacokinetic analysis was conducted with an analysis dataset containing 8180 observed elotuzumab serum concentrations from 440 out of 441 (99.8%) patients with MM who received elotuzumab across the five clinical trials described; this included all patients for whom at least one quantifiable elotuzumab serum concentration value was available.

Exposure–response analyses to characterize efficacy and safety were conducted using analysis datasets from 115 patients with RRMM who participated in ELOQUENT-3 (E-Pd, *N* = 60; Pd, *N* = 55). The exposure–response analysis datasets only included patients with available summary measures of elotuzumab exposure from the population pharmacokinetic analysis. Elotuzumab exposures in the Pd arm were imputed to be zero. Sensitivity analyses were conducted for exposure–response of efficacy and safety by excluding patients in the Pd arm from the datasets and evaluating the impact on parameter estimates.

Missing dose time was imputed using nominal values. Dose records with missing information that could strongly affect the sample, elotuzumab serum samples below the limit of quantification, and missing elotuzumab serum concentrations or those with missing pharmacokinetic sample date/time or dose data, were all excluded from the population pharmacokinetic analysis. Missing covariates were imputed based on the median (continuous) or mode (categorical) of covariates.

### Population pharmacokinetic analysis

The starting point for developing the population pharmacokinetic model was a previously established final model that included the effects of significant covariates on elotuzumab pharmacokinetic parameters [18]. The model had two compartments with parallel linear and Michaelis–Menten elimination, and additional target-mediated elimination from the periphery where the target was assumed to be non-renewable (Supplementary Figure S1). The model was updated to account for an additional effect of concomitant Pd administration on elotuzumab’s non-specific clearance and peripheral target-mediated elimination [18]. Prior elotuzumab population pharmacokinetic analyses found that target-mediated elimination decreased with a decrease in serum M-protein concentrations over time; therefore, the model was also updated to evaluate the effect of time-varying serum M-protein, rather than concentrations at baseline [24]. Linear interpolation of measured M-protein concentrations was used to assign an M-protein concentration at any time during treatment.

The choice of parameter–covariate relationships was based on findings from prior analyses [18]. Concomitant Ld or Pd was hypothesized to potentially impact on non-specific clearance based on prior analysis. The following covariate–pharmacokinetic parameter relationships were tested in the updated full model: baseline body weight and concomitant Ld or Pd on non-specific clearance; baseline body weight, sex, race (Asian versus non-Asian), and baseline β-2-micoglobulin on central volume of distribution; time-varying serum M-protein concentration on the maximum rate of Michaelis–Menten elimination (V_max_); concomitant Ld or Pd on the second-order elimination rate constant of the drug–target complex in the periphery (K_int_); and baseline body weight on the peripheral volume of distribution. Consistent with previous population pharmacokinetic analyses, covariates that had an effect on pharmacokinetic parameters that was greater than ± 20% were considered potentially clinically important [18, 24].

The updated population pharmacokinetic model was evaluated with visual predictive check (VPC) plots. The VPCs were performed with patients grouped by study and dose level and stratified by influential covariates. The VPC compared the median, and 5th and 95th percentiles of the observed concentration–time data of patients in each group with the 90% prediction interval of the corresponding statistics from 1000 simulations. The 95% CIs were obtained from standard errors of nonlinear mixed-effects model parameter estimates by observing the 2.5th and 97.5th percentiles of the resulting parameter distributions. The full model control file is described in the Supplementary Methods.

Elotuzumab concentration–time profiles and exposure measures were then simulated from the full model for patients who had individual elotuzumab pharmacokinetic parameters available. Summary measures of exposures were compared between elotuzumab dosing regimens when co-administered with Ld or Pd. Daily C_avg_ was simulated using the patient’s actual dosing history and dividing the daily predicted area under the concentration–time curve by the time interval of one day from the first day of treatment to the last day of dosing plus 60 days. The population pharmacokinetic analysis was performed using a nonlinear mixed-effects modeling program that employed the Monte Carlo expectation–maximization estimation method with importance sampling assisted by mode a posteriori (NONMEM version 7.3, ICON Development Solutions, Hanover, MD, USA) [25], and graphics were prepared using R software (version 3.2.5).

### Exposure–response analysis

Daily time-varying average concentration (daily C_avg_) was used as the measure of elotuzumab exposure in the exposure–response analyses. Daily C_avg_ closely approximates a patient’s continuously varying concentration–time profile because decreases in concentration over 24 h are relatively small compared with decreases over a dosing interval, particularly for a monoclonal antibody with a long half-life. Daily C_avg_ represents differences in peak, trough, and overall concentrations produced over the course of treatment, including changes in the elotuzumab concentration–time profile with the transition from QW to Q4W dosing, as well as the effect of accumulation with repeated dosing. In addition, daily C_avg_ accounts for dose interruptions or delays because it is derived using each patient’s actual dosing history.

The covariates included in the exposure–response analyses of PFS and time to the first occurrence of grade 3 + AEs were either identified as significant in previous exposure–response analyses or were not previously assessed but are of further clinical interest.

#### Exposure–response analysis: PFS

PFS was selected as the measure of efficacy because it was the primary endpoint in ELOQUENT-3. The relationship between elotuzumab daily C_avg_ and PFS was described by a semi-parametric Cox proportional-hazards (CPH) model that included assessments of the effects of patient-specific and disease-specific covariates on this relationship. The time-varying hazard of the risk of PFS was expressed as:

$$\lambda \left( t \right)\; = \;\lambda_{0} \left( t \right)\exp \left( {{\varvec{\beta}}^{T} {\varvec{X}}_{i} } \right)$$

where $\;\lambda_{0} \left( t \right)$ is the baseline hazard function and ${\varvec{X}}_{i}$ is a vector of predictor variables, including daily C_avg_ and other covariates. The parameter vector ${\varvec{\beta}}$ is estimated by maximum partial likelihood.

The CPH model was developed in two stages: a full model characterizing the exposure–PFS relationship while including all pre-specified covariates of interest, and a final model obtained by eliminating covariates one at a time to select the CPH model with the lowest goodness-of-fit according to the Bayesian Information Criterion. A covariate was not considered to have a statistically significant effect if the 95% CI included unity. The covariates included in the full CPH model were baseline lactate dehydrogenase, baseline beta-2 microglobulin, baseline urine M-protein, cytogenetic mutation of *t*(4:14), prior stem cell transplantation, time from diagnosis, baseline κ light chains, baseline *λ* light chains, and refractory status to lenalidomide and/or a PI. The final CPH model was evaluated with VPC plots comparing the 90% model-predicted cumulative time-to-event distributions for PFS with the corresponding distribution determined by non-parametric Kaplan–Meier analysis. The CPH model-predicted event probability of each patient, which was used to simulate the occurrence of events and subsequently calculate the cumulative time-to-event distribution. There were 1000 such simulations performed to construct the 90% prediction intervals of the distribution. The full model was also used to predict the HR and 95% CI of PFS at the 5th, 50th, and 95th percentiles of the average concentration after the first elotuzumab dose (C_avg1_) relative to the reference value to assess the impact of elotuzumab exposure on risk of disease progression or death. The exposure–response of PFS analysis, graphics, and presentation of data were performed using R software (version 3.2.5).

#### Exposure–response analysis: Grade 3 + AEs

Time to the first occurrence of grade 3 + AEs of any cause was selected as a clinically meaningful safety endpoint. The relationship between elotuzumab daily C_avg_ and grade 3 + AEs was also described by a semi-parametric CPH model that assessed the effects of pre-specified covariates on the relationship. The time-varying hazard of the risk of grade 3 + AEs was expressed by the same model equation as described in the previous section for PFS. It was developed in two stages as full and final model as described for the exposure–response of PFS analysis. Similarly, the final model was evaluated by VPC plots using the methods used to assess the CPH model for PFS. The covariates included in the full CPH model were Eastern Cooperative Oncology Group (ECOG) performance status and baseline beta-2 microglobulin. The full model was also used to predict the HR and 95% CI of grade 3 + AEs at the 5th, 50th, and 95th percentiles of C_avg1_ relative to the reference value to assess the impact of elotuzumab exposure on risk of developing grade 3 + AEs. The exposure–response of grade 3 + AEs analysis, graphics, and presentation of data were performed using R software (version 3.2.5).

## Results

### Population pharmacokinetic analysis

Consistent with the earlier population pharmacokinetic analysis [18, 24], elotuzumab pharmacokinetics were well characterized by a two-compartment model with zero-order IV infusion and first-order elimination, with parallel linear and Michaelis–Menten elimination and additional target-mediated elimination from the periphery. As shown in Figure captions

**Fig. 1:**
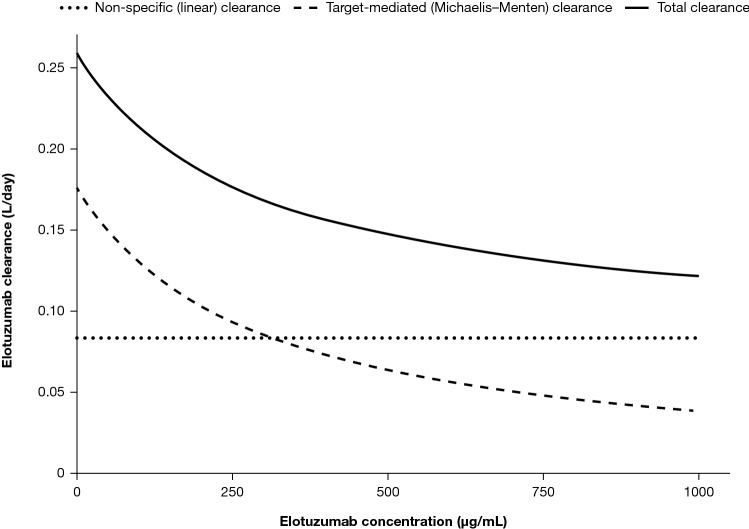
Elimination of elotuzumab from the central compartment using non-specific (linear) and target-mediated (Michaelis–Menten or non-linear) components

Figure 1, elotuzumab’s non-specific clearance was linear and did not change with time, whereas target-mediated or Michaelis–Menten clearance decreased with increasing elotuzumab concentrations, which is consistent with nonlinear pharmacokinetics. Parameter estimates from the full population pharmacokinetic model are provided in Supplementary Table S2 and the impact of the covariates on elotuzumab pharmacokinetics is presented in Supplementary Fig. S2. Briefly, the following patient-specific and disease-specific covariates were statistically significant in the model: baseline body weight and co-administration with Pd or Ld on non-specific CL; baseline body weight, baseline beta-2 microglobulin, sex, and race on the central volume of distribution; baseline body weight on peripheral volume of distribution; time-varying serum M-protein on V_max_; and co-administration of Pd or Ld on K_int_.

The point estimates for the effects of sex, race, and baseline beta-2 microglobulin on the volume of the central compartment (VC) were within ± 20% of the reference value and were therefore not considered clinically important. Non-specific clearance of elotuzumab increased in patients with a higher baseline body weight [clearance in patients weighing 50.6 kg (5th percentile) and 105 kg (95th percentile) was ~ 41% lower and ~ 55% higher, respectively, compared with patients weighing 75 kg (reference value)]. V_max_ increased with increasing serum M-protein, resulting in lower elotuzumab exposures in patients with higher serum M-protein concentrations. Co-administration of Pd resulted in 19% and 51% decreases in elotuzumab clearance and K_int_, respectively, relative to co-administration with Ld. The full model was extensively evaluated using diagnostic plots and predictive check procedures and was deemed acceptable for predicting individual elotuzumab exposures as well as for model-based simulation.

**Fig. 2:**
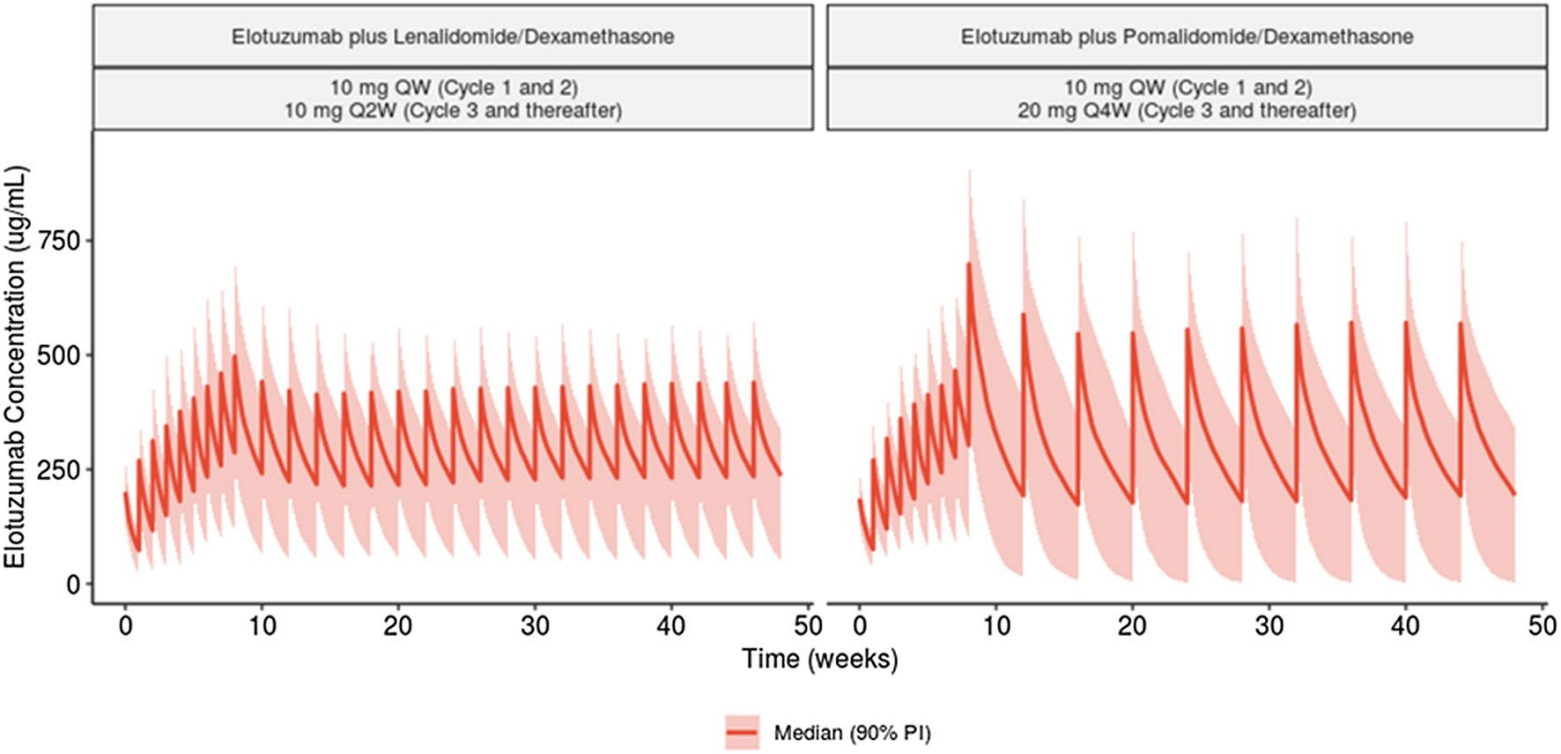
Predicted elotuzumab concentration–time course following elotuzumab 10 mg/kg IV QW administration for Cycle 1 and 2, followed by 10 mg/kg IV Q2W (in combination with lenalidomide/dexamethasone) or 20 mg/kg IV Q4W (in combination with pomalidomide and dexamethasone) for subsequent cycles. The red lines represent median (5th and 95th percentiles) of elotuzumab concentration distribution. PI, prediction interval; QW, once every week Q2W, every 2 weeks; Q4W, every 4 weeks

Model-predicted mean serum concentration–time profiles following elotuzumab 10 mg/kg IV QW for two 28 day cycles followed by 10 mg/kg IV Q2W plus Ld or 10 mg/kg IV QW for two 28 day cycles followed by 20 mg/kg IV Q4W plus Pd are shown in Fig. 2. Model-predicted geometric mean C_avgSS_ was similar between the two dosing regimens while geometric mean C_minSS_ and geometric mean C_maxSS_ are 31% lower and 38% higher, respectively, for the 20 mg/kg IV Q4W maintenance dosing regimen (Table 2). Regardless of the IMiD backbone and elotuzumab maintenance dosing regimen, peripheral concentrations of elotuzumab decreased similarly over time suggesting that steady-state exposures were driven primarily by non-specific CL. The model-derived mean effective half-life of elotuzumab was 37.1 days in patients receiving E-Pd and 31.2 days in patients receiving E-Ld. Regardless of IMiD backbone or maintenance dosing regimen, steady-state elotuzumab exposures were achieved by approximately 16 weeks.

**Table 2:** Predicted geometric mean (CV) of exposure measures by co-administration following administration of recommended dosing regimens of elotuzumab

	C_avg1_ (μg/mL) GeoMean (CV)	C_min1_ (μg/mL) GeoMean (CV)	C_max1_ (μg/mL) GeoMean (CV)	C_avgSS_ (μg/mL) GeoMean (CV)	C_minSS_ (μg/mL) GeoMean (CV)	C_maxSS_ (μg/mL) GeoMean (CV)
Concomitant administration						
Lenalidomide/dexamethasone^a^ (*N* = 349)	114 (0.262)	63.4 (0.386)	195 (0.222)	260 (0.355)	179 (0.428)	394 (0.288)
Pomalidomide/dexamethasone^b^ (*N* = 60)	113 (0.232)	69.7 (0.337)	185 (0.194)	266 (0.426)	124 (0.585)	543 (0.275)

^a^Elotuzumab 10 mg/kg IV QW administration for Cycles 1 and 2, followed by 10 mg/kg IV Q2W for subsequent cycles [18]

^b^Elotuzumab 10 mg/kg IV QW administration for Cycles 1 and 2, followed by 20 mg/kg IV Q4W for subsequent cycles [13]

*C*_*avg1*_ time-averaged concentration after the first elotuzumab dose, *C*_*avgSS*_ time-averaged concentration at steady state, *C*_*max1*_ peak concentration after the first elotuzumab dose, *C*_*maxSS*_ peak concentration at steady state, *C*_*min1*_ trough concentration after the first elotuzumab dose, *C*_*minSS*_ trough concentration at steady state, *CV* coefficient of variation, *GeoMean* geometric mean

### Exposure–response analysis: PFS

The parameter estimates for the full CPH model expressed as HRs and 95% CIs for continuous and categorical covariates are summarized in Supplementary Table S3. The covariates with a significant effect on the risk of disease progression or death (95% CI does not include unity) were daily C_avg_, baseline lactate dehydrogenase, and baseline beta-2 microglobulin. The 95% CI of the effect for all other covariates in the full CPH model included unity, indicating a lack of effect on the risk of disease progression or death. The risk of disease progression or death appeared to decrease with increasing elotuzumab daily C_avg_.

**Fig. 3:**
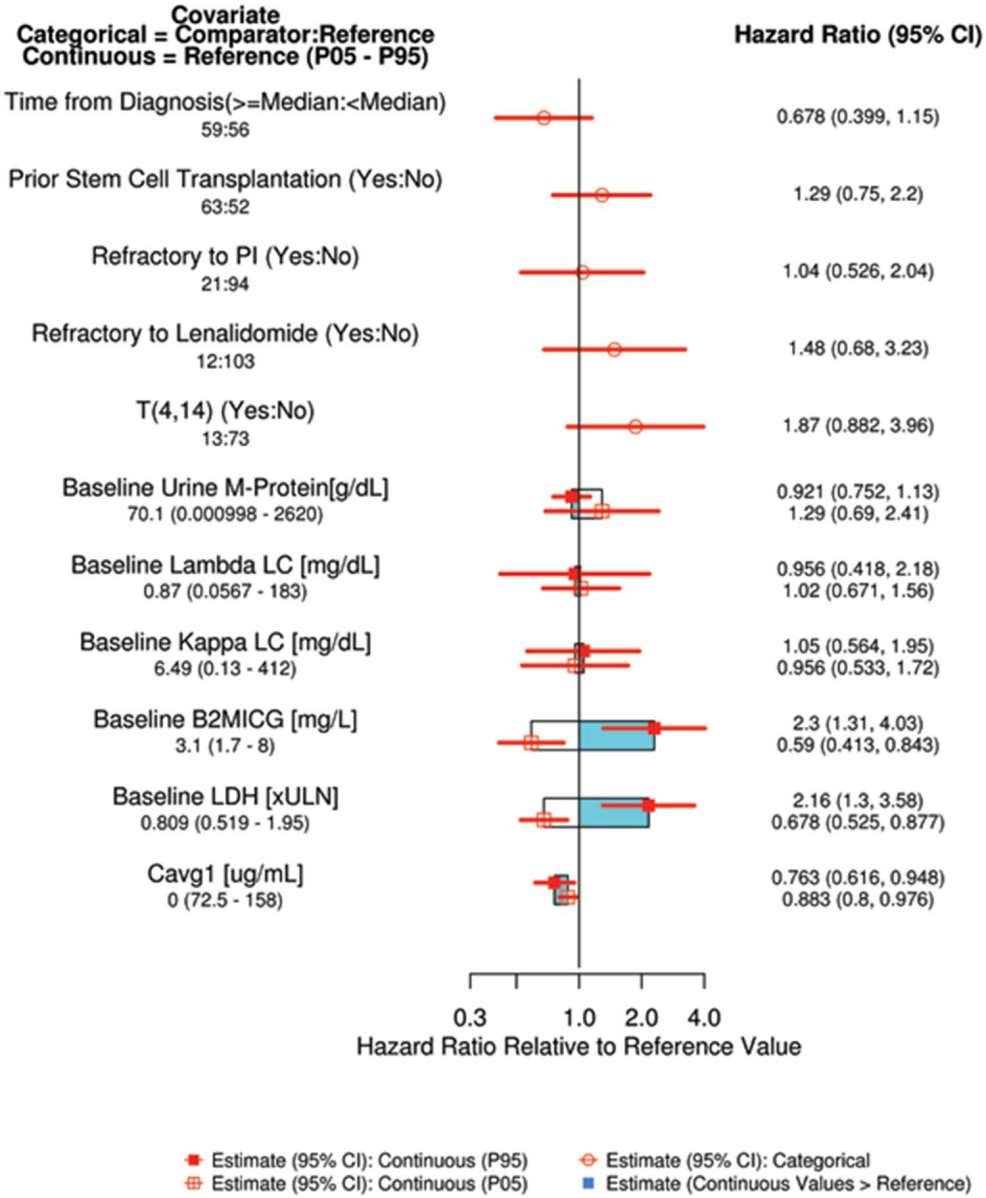
Predictors of the hazard ratio of full exposure–response model of efficacy (progression-free survival). Continuous covariate effects (95% CI) at the 5th/95th percentiles of the covariate are represented by the end of horizontal boxes (horizontal lines). Open/shaded area of boxes represents the range of covariate effects from the median to the 5th/95th percentile of the covariate. Categorical covariate effects (95% CI) are represented by open circles (horizontal lines). *B2MICG* serum beta-2 microglobulin, *C*_*avg1*_ time-averaged concentration after the first elotuzumab dose, *CI* confidence interval, *LC* light chain, *LDH* lactate dehydrogenase, *PI* prediction interval, *ULN* upper limit of normal

The magnitude of the covariate effects on PFS is presented in Fig. 3. Given that daily C_avg_ does not reflect a single exposure time point for an individual patient, average concentration after the first dose (C_avg1_) was used to visualize the magnitude of the exposure effect on PFS. Risk of disease progression or death increased for patients with higher baseline lactate dehydrogenase, higher baseline beta-2 microglobulin, who were refractory to lenalidomide, had a prior stem cell transplantation, and who had the *t*(4,14) chromosomal abnormality. Risk of disease progression or death decreased for patients whose time from diagnosis was longer than the median time in ELOQUENT-3 and who had higher C_avg1_. After stepwise backward elimination based on Bayesian Information Criterion, the covariates retained in the final CPH model were daily C_avg_, baseline lactate dehydrogenase, and baseline beta-2 microglobulin. VPC plots of the final CPH model stratified by treatment (Supplementary Figure S3) indicated that the model-predicted PFS was in good agreement with the observed PFS. The sensitivity analysis excluding patients in the Pd arm showed that the effect of time-varying C_avg_ on the HR and 95% CI of PFS was comparable to that observed in the main analysis, indicating no significant impact of including control patients in the exposure–response analysis (Supplementary Table S5).

The HR of PFS at the 95th percentile relative to the 5th percentile of C_avg1_ was predicted from the final CPH model. The risk of disease progression or death decreased with increasing elotuzumab exposure (HR 0.853, 95% CI 0.765–0.951).

### Exposure–response analysis: grade 3 + AEs

**Fig. 4:**
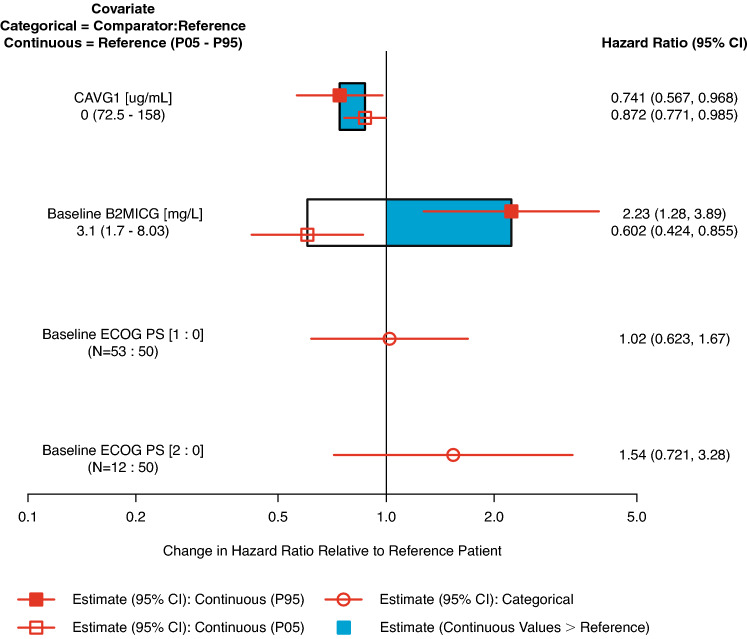
Predictors of the hazard ratio of full exposure–response model of safety (grade 3 + adverse events). Continuous covariate effects (95% CI) at the 5th/95th percentiles of the covariate are represented by the end of horizontal boxes (horizontal lines). Open/shaded area of boxes represents the range of covariate effects from the median to the 5th/95th percentile of the covariate. Categorical covariate effects (95% CI) are represented by open circles (horizontal lines). *B2MICG* serum beta-2 microglobulin, *C*_*avg1*_ time-averaged concentration after the first elotuzumab dose, *CI* confidence interval, *ECOG PS* Eastern Cooperative Oncology Group performance status, *P05–P95* 5th/95th percentiles

The parameter estimates for the full CPH model expressed as a HR and 95% CI for continuous and categorical covariates are summarized in Supplementary Table S4 and the magnitude of these effects is shown in Fig. 4. The point estimates for daily C_avg_ were negative suggesting that the risk of developing grade 3 + AEs decreases with increasing elotuzumab exposures within the range studied. The risk of grade 3 + AEs increased with elevated baseline beta-2 microglobulin but did not increase in patients with a poor ECOG performance status (ECOG 1 or 2 versus 0) as the 95% CIs included unity. As the aim of the analysis was to assess the relationship between theh first occurrence of grade 3 + AEs and exposure, daily C_avg_ was retained in the model and was not subjected to backward elimination. After stepwise backward elimination, the only covariate retained in the final CPH model in addition to daily C_avg_ was baseline beta-2 microglobulin. A VPC plot of the final CPH model stratified by treatment (Supplementary Figure S4) indicated good agreement between the model predicted and observed incidence of grade 3 + AEs.

The HR of grade 3 + AEs at the 95th percentile relative to the 5th percentile of C_avg1_ was predicted from the final CPH model. The risk of grade 3 + AEs did not increase with increasing elotuzumab exposure (HR 0.845, 95% CI 0.734–0.980). The sensitivity analysis excluding patients in the Pd arm showed that the effect of time-varying C_avg_ on the HR and 95% CI for time to grade 3 + AEs was comparable to that observed in the main analysis, indicating no significant impact of including control patients in the exposure–response analysis (Supplementary Table S6).

## Discussion

A pooled population pharmacokinetic approach with data from five clinical studies that included elotuzumab as monotherapy or in combination with Ld or Pd in various MM disease settings was used to assess the pharmacokinetics of elotuzumab in patients with MM. The population pharmacokinetic model was updated to evaluate the effects of concomitant Pd administration as well as time-varying serum M-protein on elotuzumab pharmacokinetics. The elotuzumab exposures determined as part of this analysis were evaluated in exposure–response analyses for their relationship with efficacy and safety in patients with RRMM from ELOQUENT-3.

Consistent with prior population pharmacokinetic analyses [18, 24], elotuzumab concentration–time data were well described by a two-compartment model with parallel linear and Michaelis–Menten elimination and additional target-mediated elimination from the peripheral compartment with a non-renewable amount of target. The estimated pharmacokinetic parameters and covariate effects were in good agreement with the previously conducted analysis of elotuzumab pharmacokinetics in patients with RRMM [18]. In the current analysis, concomitant Pd resulted in an approximately 19% and 51% decrease in elotuzumab non-specific clearance and K_int_, respectively, relative to concomitant Ld. However, model-predicted geometric mean C_avgSS_ was similar between the two IMiD backbones and maintenance dosing regimens (260 μg/mL with Ld versus 266 μg/mL with Pd). As the safety and efficacy of elotuzumab plus Pd were previously demonstrated in ELOQUENT-3 [17], these pharmacokinetic findings are unlikely to be clinically meaningful.

Unlike the prior population pharmacokinetic analysis that used baseline serum M-protein concentrations, the updated analysis included time-varying serum M-protein to describe elotuzumab’s target-mediated drug disposition. The prior analysis identified a decrease in elotuzumab elimination with time, a decrease in elotuzumab’s target-mediated elimination with a decrease in serum M-protein concentrations, and a decrease in serum M-protein concentrations over time. Collectively, these findings support the inclusion of time-varying serum M-protein in the current model, which better represents elotuzumab’s clearance over time in patients with MM. The effect of time-varying serum M-protein on V_max_ was approximately 52% higher in the current analysis versus the earlier analysis utilizing baseline serum M-protein only [18]. M-protein is secreted by myeloma cells; therefore, elevated serum M-protein concentrations may reflect a higher tumor burden and more available target, thus facilitating faster target-mediated elimination of elotuzumab. Serum M-protein concentrations declined over time in the majority of patients, leading to relatively minor differences in steady-state exposure between patients with widely different baseline serum M-protein levels.

In ELOQUENT-3, a less frequent elotuzumab dosing regimen of 20 mg/kg IV Q4W starting at Cycle 3 was evaluated. Geometric mean C_minSS_ was 31% lower and C_maxSS_ was 38% higher, compared with 10 mg/kg IV Q2W plus Ld; however, geometric mean C_avgSS_ was similar between the two regimens. The 20 mg/kg IV Q4W regimen is more convenient for patients due to the less frequent administration schedule. In addition, ELOQUENT-3 demonstrated efficacy and safety with elotuzumab plus Pd compared with Pd alone.

Among patients with MM who were refractory to lenalidomide and a PI, E-Pd resulted in statistically significant longer PFS than Pd alone (10.3 versus 4.7 months) [17]. The exposure–response analysis assessing PFS suggested that clinical benefit with E-Pd compared with Pd was attained even in patients with the lowest (ie, 5th percentile) elotuzumab exposures following 10 mg/kg IV QW for the first two 28 day cycles followed by 20 mg/kg IV Q4W thereafter. The risk of disease progression or death appeared to decrease with increasing elotuzumab exposures; however, the magnitude of the difference in the HRs at the 5th and 95th percentiles of exposure relative to patients in the control arm (Pd alone) was relatively small (0.883 and 0.763, respectively). As described previously, a higher tumor burden as measured by serum M-protein was associated with faster target-mediated elimination and thus lower elotuzumab exposures. Given that a range of dosing regimens was not evaluated in ELOQUENT-3, and exposure and clearance are correlated, any apparent relationship between elotuzumab exposure and risk of disease progression or death may be confounded by the patient’s disease status (eg, serum M-protein and other disease-related factors). This finding is consistent with reports highlighting a confounded exposure–response relationship including clearance of monoclonal antibody immune checkpoint inhibitors that target programmed death-1 [26, 27]. Therefore, no causal relationship can be established between low elotuzumab exposures and higher risk of disease progression or death based on the current exposure–response of efficacy analysis. The results from a phase 2 study of E-Ld, in which patients were randomized to elotuzumab 10 mg/kg QW for Cycles 1 and 2 and 20 mg/kg IV Q2W in subsequent cycles, suggest that higher steady-state exposures with 20 mg/kg IV Q2W did not seem to reduce the risk of disease progression or death more than 10 mg/kg IV Q2W [28], indicating that both dosing regimens achieved maximum efficacy [28].

Elotuzumab had an acceptable safety profile over the exposure range achieved with the dosing regimen evaluated in ELOQUENT-3, and higher exposures were not associated with an increased risk of grade 3 + AEs. Interestingly, the risk of grade 3 + AEs was lower in E-Pd–treated patients compared with Pd-treated patients. A lower risk of grade 3 + AEs in patients with higher exposures might be indicative of an improvement in the health of patients who better tolerate elotuzumab and can remain on therapy for a longer period of time.

The clinical implications of these results are minimal, and low elotuzumab exposures over the approved dose range are not causal of higher risk of progression/death. Indeed, clinically significant differences have not been observed in the pharmacokinetics of elotuzumab based on age, sex, race, baseline lactate dehydrogenase, albumin, renal or mild hepatic impairment, and coadministration with Ld or Pd [29].

In summary, this work is the first report on the population pharmacokinetics of elotuzumab across several studies including ELOQUENT-3, as well as exposure–response relationships in patients with RRMM who were co-administered Pd. No clinically relevant differences in elotuzumab exposures were observed between patients who received E-Pd or E-Ld. Although the elotuzumab dosing regimen of 20 mg/kg IV Q4W beginning at Cycle 3 evaluated in ELOQUENT-3 produced slightly higher C_maxSS_ and lower C_minSS_, respectively, compared with the 10 mg/kg IV Q2W regimen evaluated in ELOQUENT-2 (with Ld), C_avgSS_ was similar between the two regimens. Across the range of exposures achieved with the elotuzumab dosing regimen in ELOQUENT-3, increasing elotuzumab daily C_avg_ prolonged PFS in patients with RRMM without increasing the risk of first occurrence of grade 3 + AEs.

## Supplementary Information

Below is the link to the electronic supplementary material.

Supplementary file1 (PDF 836 KB)

## Data Availability

Bristol Myers Squibb’s policy on data sharing may be found at https://www.bms.com/researchers-and-partners/clinical-trials-and-research/disclosure-commitment.html.
