# Supplementary material for: Population pharmacokinetic and exposure–response analyses of elotuzumab plus pomalidomide and dexamethasone for relapsed and refractory multiple myeloma
Source: Cancer Chemother Pharmacol. 2021 Nov 26;89(1):129–40. doi: 10.1007/s00280-021-04365-4 (PMC8739320; doi:10.1007/s00280-021-04365-4)
Supplement: Supplementary file 1 — Supplementary file1 (PDF 836 KB) [file 280_2021_4365_MOESM1_ESM.pdf]

Supplementary materials

Methods

Population pharmacokinetic analysis: NONMEM control file of full covariate model

```
$SIZES LIM1=70000

$PROBLEM final model (previous final model plus Lendex/PomDex on CL and KINT)

$INPUT C=DROP,STUDY,USUBJID=DROP,ID,TIME,AMT,RATE,DUR=DROP,CONC,DV,MDV,EVID,TAD,AGE,WT,ALT=DROP,
TITER,HT=DROP,CREAT=DROP,ALP=DROP,AST=DROP,BILI=DROP,LDH_Z,MCPROT_Z,ALB,B2MICG_Z,BSA=DROP,BMI=DROP,
CRCL=DROP,CRCLN=DROP,GFR_Z,SEXF,RACEN,ECOG101,ADA,NAB,NABADA,ARM=DROP,DOSE=DROP,RENAL,HEP_Z,
LDOS=DROP,CODE=DROP,IMPDOS=DROP,IMPDAT=DROP,IMPTIM=DROP,NTAD=DROP,VISITNUM=DROP,CDATE=DROP,
CTIME=DROP,DAY=DROP,CNTR=DROP,HISP=DROP,CYCLE=DROP,CDAY=DROP,NTIME=DROP,JAPAN,COUNTRY=DROP,SEX=D
ROP,RACEC=DROP,T1,TITER1,T2,TITER2,TMCPROT_Z,TMCPROTUR=DROP,TALB=DROP,LINEF=DROP

$DATA ../data/ppk.csv IGNORE=C IGNORE=(CODE.EQ.12)

$SUBROUTINE ADVAN13 TOL=9

$MODEL
COMP=(DRUG) ;Central Compartment (Free Drug)
COMP=(Periph) ;Peripheral Drug Compartment
COMP=(Periph) ;Target Compartment

$PK

MVAL=-99
; impute value for missing of LDH
        LDH_R= 199
        LDH=LDH_Z
IF(LDH.EQ.MVAL) LDH= LDH_R

; impute value for missing of B2MICG
        B2MICG_R= 0.32
        B2MICG=B2MICG_Z
IF(B2MICG.EQ.MVAL) B2MICG= B2MICG_R


; impute value for missing of HEP
        HEP_R= 0
```

```
    HEP=HEP_Z
IF(HEP.EQ.MVAL) HEP= HEP_R

; impute value for missing of GFR
GFR_R= 75.39
GFR=GFR_Z
IF(GFR.EQ.MVAL) GFR= GFR_R

; impute value for missing of MCPROT
MCPROT_R= 2.05
MCPROT=MCPROT_Z
IF(MCPROT.EQ.MVAL) MCPROT= MCPROT_R

; impute value for missing of TMCPROT
TMCPROT_R= 2.05
TMCPROT=TMCPROT_Z
IF(TMCPROT.EQ.MVAL) TMCPROT= TMCPROT_R

STOTHER = 0
IF(STUDY.NE.204004) STOTHER = 1

VWT = (WT/75)

ASIAN = 0
IF(RACEN.EQ.3) ASIAN = 1

HEPA = 0
IF(HEP.GT.0.5) HEPA = 1

ECOG1 = 0
IF(ECOG101.GT.0.5) ECOG1 = 1

ECOG2 = 0
IF(ECOG101.GT.1.5) ECOG2 = 1

B2MICG1 = 0
IF(B2MICG.GE.0.2) B2MICG1 = 1
```

```
B2MICG2 = 0

IF(B2MICG.GE.0.35) B2MICG2 = 1

LENDEX = 0
IF(STUDY.NE.204011) LENDEX = 1

POMDEX = 0
IF(STUDY.EQ.204125) POMDEX = 1

VCOV = THETA(17)*SEXF+THETA(18)*ASIAN
CLCOV = (LENDEX-1)*THETA(19)+POMDEX*THETA(20)
KINTCOV = (LENDEX-1)*THETA(21)+POMDEX*THETA(22)

CLCOV =
CLCOV+THETA(24)*SEXF+THETA(25)*ASIAN+LOG(AGE/65)*THETA(26)+LOG(GFR/100)*THETA(27)+LOG(LDH/200)*THETA(28)
CLCOV = CLCOV+THETA(29)*HEPA+LOG(ALB/3.5)*THETA(30)+THETA(31)*ECOG1+THETA(32)*ECOG2
CLCOV = CLCOV+THETA(33)*B2MICG1+THETA(34)*B2MICG2
VCOV = VCOV+THETA(35)*B2MICG1+THETA(36)*B2MICG2

MU_1 = THETA(1)+ THETA(9)*LOG(VWT)+CLCOV
MU_2 = THETA(2)+THETA(10)*LOG(VWT)+VCOV
MU_3 = THETA(3)+THETA(11)*LOG(VWT)
MU_4 = THETA(4)+THETA(12)*LOG(VWT)
MU_5 = THETA(5)
MU_6 = THETA(6)+KINTCOV
MU_7 = THETA(7)
MU_8 = THETA(8)

CL = EXP(MU_1+ETA(1)) ;1 CL [L/day]
VC = EXP(MU_2+ETA(2)) ;2 Vc [L]
Q = EXP(MU_3+ETA(3)) ;3 Q [L/day]
VP = EXP(MU_4+ETA(4)) ;4 Vd [L]
RMAX = EXP(MU_5+ETA(5))
KINT = EXP(MU_6+ETA(6))/1000
VMAX = EXP(MU_7+ETA(7))*EXP(THETA(23)*TMCPROT)
KM = EXP(MU_8+ETA(8))

K10=CL/VC
K12=Q/VC
```

```
K21=Q/VP

SDL = THETA(13)
SDH = THETA(14)
SD50 = THETA(15)

A_0(3)=RMAX

$DES
CONC1 = A(1)/VC
DADT(1)=-K12*A(1)+K21*A(2)-K10*A(1)-VMAX*A(1)/(CONC1+KM)
DADT(2)= K12*A(1)-K21*A(2)-KINT*A(2)*A(3)
DADT(3)=-KINT*A(2)/VP*A(3)

$ERROR
TY= A(1)/VC
LTY=-2.5
IF(TY.GT.0) LTY=LOG(TY)
W = (SDL-(SDL-SDH)*TY/(SD50+TY))*THETA(16)**STOTHER*EXP(ETA(9))
Y=LTY+W*EPS(1)

IRES = DV-LTY
IWRES=IRES/W

IPRED = Y

$THETA
(-4,-2.53,0) ; 1 CL (L/day)
(0,1.346,4) ; 2 VC (L)
(-2,-0.507,1) ; 3 Q (L/day)
(0,0.679,3) ; 4 VP (L)
(4,6.81,10) ; 5 RMAX
(-4,-1.53,0) ; 6 KINT
(0,2.50,4) ; 7 VMAX
(-4,5.71,6) ; 8 KM

(0,1.18) ; 9 WT to CL
(0,0.387) ;10 WT to V
```

(0,0.75) FIXED ;11 WT to Q

(0,0.792) ;12 WT to VP

(0,2.64) ;13 SDL

(0,0.091) ;14 SDH

(0,6.13) ;15 SD50

(0,0.72,2) ;16 STOTHER error

(-1,-0.19,0) ;17 female to VC

(-1,-0.16,0) ;18 Asian to VC

(-1,-0.32,0) ;19 LenDex to CL

(-1,-0.21,0) ;20 PomDex to CL

(0,2.36,5) ;21 LenDex to Kint

(-2,-0.72,0) ;22 PomDex to Kint

(0,0.26,1) ;23 TMCPROT to VMAX

0 FIXED ;24 female to CL

0 FIXED ;25 Asian to CL

0 FIXED ;26 AGE to CL

0 FIXED ;27 GFR to CL

0 FIXED ;28 LDH to CL

0 FIXED ;29 HEP > 1 to CL

0 FIXED ;30 ALB to CL

0 FIXED ;31 ECOG101 > 0.5 to CL

0 FIXED ;32 ECOG101 > 1.5 to CL

0 FIXED ;33 B2MICG >= 2 to CL

0 FIXED ;34 B2MICG >= 3.5 to CL

0 FIXED ;35 B2MICG >= 2 to V

(-2,0.01,2) ;36 B2MICG >= 3.5 to V

$OMEGA

0.166 ; 1 CL

0.035 ; 2 VC

0.552 ; 3 Q

0.137 ; 4 VP

0.161 ; 5 RMAX

1.390 ; 6 KINT

0.0001 FIX ; 7 VMAX

```
0.317 ; 8 KM
0.182 ; 9 EPS

$SIGMA
1 FIXED

$EST METHOD=IMPMAP INTERACTION RANMETHOD=P EONLY=0 NITER=3000 ISAMPLE=500 PRINT=1
NOABORT NSIG=2 SIGL=7 CTYPE=3 GRD=TG(1-12):TS(13-16):TG(17-22):TD(23):TG(24-36)
NOTHETABOUNDTEST NOOMEGABOUNDTEST NOSIGMABOUNDTEST

$EST METHOD=IMPMAP INTERACTION RANMETHOD=P EONLY=1 NITER=20 ISAMPLE=1000 PRINT=1
NSIG=3 SIGL=9 NOTHETABOUNDTEST NOOMEGABOUNDTEST NOSIGMABOUNDTEST NOABORT
GRD=TG(1-12):TS(13-16):TG(17-22):TD(23):TG(24-36) MSFO=FILE.MSF

$COV PRINT=E UNCONDITIONAL

$TABLE ID TIME TAD EVID MDV CONC TY LTY IPRED IWRES CWRES CWRESI ECWRES NPDE NPD
CL VC Q VP RMAX KINT VMAX KM ETA1 ETA2 ETA3 ETA4 ETA5 ETA6 ETA7 ETA8 ETA9
WT TMCPROT AGE GFR LDH ALB SEXF ASIAN LENDEX POMDEX HEPA ECOG1 ECOG2 B2MICG1 B2MICG2
TITER T1 T2 TITER1 TITER2 ADA NAB NABADA
NOPRINT ONEHEADER FILE=tab1

$TABLE ID TIME TAD EVID MDV CONC TY LTY IPRED IWRES CWRES CWRESI
NOPRINT ONEHEADER FILE=sdtab1

$TABLE ID TIME TAD EVID MDV CL VC Q VP RMAX KINT VMAX KM
ETA1 ETA2 ETA3 ETA4 ETA5 ETA6 ETA7 ETA8 ETA9
NOPRINT ONEHEADER FILE=patab1

$TABLE ID TIME TAD EVID MDV WT TMCPROT MCPROT AGE GFR LDH ALB
NOPRINT ONEHEADER FILE=cotab1
$TABLE ID TIME TAD EVID MDV SEXF ASIAN LENDEX POMDEX HEPA ECOG1 ECOG2 B2MICG1 B2MICG2
NOPRINT ONEHEADER FILE=catab1
```

Supplementary Table S1 Summary of baseline covariates for subjects in the exposure–response analysis of efficacy and safety dataset (N=115)

Covariate	N (%)	Mean (SD)	Median (min, max)
Beta-2 microglobulin (mg/L)		3.68 (1.96)	3.15 (0.8, 11.5)
Baseline lactate dehydrogenase (U/L)		264 (171)	206 (93, 1160)
Natural of ratio of LDH to ULN		–0.11 (0.41)	–0.191 (–0.884, 1.45)
Serum kappa light chains (mg/dL)		83.2 (264)	6.51 (0.09, 2360)
Log serum kappa light chains		1.81 (2.63)	1.87 (–2.41, 7.77)
Serum lambda light chains (mg/dL)		47 (164)	0.87 (0.05, 1290)
Log serum lambda light chains		0.617 (2.6)	–0.139 (–3, 7.16)
Urine M-protein (mg/day)		472 (949)	69.9 (0, 5120)
Log urine M-protein		2.55 (5.13)	4.25 (–6.91, 8.54)
Chromosomal abnormality t(4:14)			
No	73 (63)		
Yes	13 (11)		
Unknown	29 (25)		
Time from disease diagnosis			
<Median (55.6 months)	56 (49)		
≥Median (55.6 months)	56 (49)		
Missing	3 (3)		
Prior stem cell transplant			
Yes	63 (55)		
No	52 (45)		
Refractory status to lenalidomide			
Yes	12 (10)		
No	100 (87)		
Missing	3 (3)		
Refractory status to protease inhibitors			
Yes	21 (18)		

Covariate	N (%)	Mean (SD)	Median (min, max)
No	92 (80)		
Missing	2 (2)		
Races			
Japanese	19 (17)		
Non-Japanese	96 (83)		
ECOG performance status			
0	50 (43)		
1	53 (46)		
2	12 (10)		

N (%) of patients with missing data: beta-2 microglobulin, 1 (1%); lactate dehydrogenase, 3 (3%); LDL to ULN, 3 (3%)

ECOG, Eastern Cooperative Oncology Group; LDH, baseline lactate dehydrogenase; SD, standard deviation; ULN, upper limit of normal

Supplementary Table S2 Parameter estimates of elotuzumab full PPK model

Parameter		Value	% RSE	95% CI[a]	CV[b]
**Structural parameters**					
CL_{REF} (L/day)	exp(θ_1)	0.0834	3.51	0.0778–0.0893	NA
VC_{REF} (L)	exp(θ_2)	4.06	1.64	3.94–4.2	NA
Q_{REF} (L/day)	exp(θ_3)	0.512	5.72	0.458–0.573	NA
VP_{REF} (L)	exp(θ_4)	1.93	3.89	1.79–2.09	NA
RMAX (μg/mL)	exp(θ_5)	849	5.19	767–940	NA
$KINT_{REF}$ (10^{-3}/day/ [μg/mL])	exp(θ_6)	0.216	9.67	0.179–0.261	NA
$VMAX_{REF}$ (μg/mL/day)	exp(θ_7)	12.2	0.577	12–12.3	NA
KM (μg/mL)	exp(θ_8)	281	4.85	255–309	NA
**Covariate effects parameters[c]**					
CL_{WT}	θ_9	1.32	9.87	1.07–1.58	NA
CL_{Mono}	exp(θ_{19})	0.825	11.7	0.657–1.04	NA
CL_{PomDex}	exp(θ_{20})	0.811	9.52	0.673–0.977	NA
VC_{WT}	θ_{10}	0.345	15	0.244–0.447	NA
VC_{SEX}	exp(θ_{17})	0.797	2.23	0.763–0.832	NA
VC_{RACE}	exp(θ_{18})	0.885	3.23	0.831–0.943	NA
$VC_{B2MICG >0.35}$	exp(θ_{36})	1.11	2.04	1.07–1.16	NA
Q_{WT}	θ_{11}	0.75 Fixed	NA	NA	NA
VP_{WT}	θ_{12}	0.696	19.1	0.435–0.958	NA
$VMAX_{MCPROT}$ $(g/dL)^{-1}$	θ_{23}	0.27	2.4	0.257–0.283	NA
$KINT_{Mono}$	exp(θ_{21})	9.78	26.8	5.79–16.5	NA
$KINT_{PomDex}$	exp(θ_{22})	0.487	22.6	0.313–0.758	NA
**Inter-individual variability parameters**					
ω^2_{CL}	Ω(1;1)	0.158	11.6	0.122–0.193	39.7%
ω^2_{VC}	Ω(2;2)	0.0361	8.67	0.03–0.0422	19%

Parameter		Value	% RSE	95% CI[a]	CV[b]
ω^2_{Q}	Ω(3;3)	0.454	13	0.338–0.57	67.4%
ω^2_{VP}	Ω(4;4)	0.133	18.7	0.0845–0.182	36.5%
ω^2_{RMAX}	Ω(5;5)	0.189	21.7	0.109–0.269	43.4%
ω^2_{KINT}	Ω(6;6)	1.69	10.4	1.35–2.04	130%
ω^2_{VMAX}	Ω(7;7)	0.0001 Fixed[d]	NA	NA	1%
ω^2_{KM}	Ω(8;8)	0.385	12.1	0.294–0.476	62%
ω^2_{ε}	Ω(9;9)	0.183	9.39	0.149–0.216	42.7%
**Intra-individual variability model parameters**					
SD_L	θ_{13}	2.46	23	1.35–3.57	NA
SD_H	θ_{14}	0.0976	5.6	0.0869–0.108	NA
SD_{50} (µg/mL)	θ_{15}	6.17	28.2	2.76–9.59	NA
$SD_{phase1,2}$	θ_{16}	0.843	5.29	0.756–0.931	NA

[a]Asymptomatic 95% CI

[b]Coefficient of variation was computed as 100*ω^2P where ωP is the square root of the corresponding variance parameter ω^2P

[c]The covariate effects were modeled as follows:

$$CL = CL_{REF}\left(\frac{WT}{75}\right)^{CL_{WT}}(CL_{Mono})^{-Mono}(CL_{PomDex})^{PomDex}$$

$$VC = VC_{REF}\left(\frac{WT}{75}\right)^{VC_{WT}}(VC_{SEX})^{SEX}(VC_{RACE})^{RACE}(VC_{B2MICG>0.35})^{B2MICG>0.35}$$

$$VP = VP_{REF}\left(\frac{WT}{75}\right)^{VP_{WT}}$$

$$Q = Q_{REF}\left(\frac{WT}{75}\right)^{Q_{WT}}$$

$$VMAX = VMAX_{REF}exp(VMAX_{MCPROT}MCPROT)$$

$$KINT = KINT_{REF}(KINT_{Mono})^{-Mono}(KINT_{PomDex})^{PomDex}$$

[d]For this parameter, variance of the random effect was estimated to be near zero, and it was fixed to a small number to allow proper performance of the Monte Carlo importance sampling EM assisted by mode a posteriori estimation (IMPMAP) method

$\omega^2\varepsilon$, variance of the random effect on the magnitude of the residual error; B2MICG, serum beta-2 microglobulin at baseline; CI, confidence interval; CL, clearance; CL_{Mono}, effect of elotuzumab monotherapy on CL; CL_{PomDex}, effect of concomitant pomalidomide/dexamethasone administration on CL; CL_{WT}, power coefficient of CL dependence on body weight; CL_{REF}, typical clearance at reference values of covariates (WT = 75 kg, co-administered lenalidomide/dexamethasone); CV, coefficient of variation; KINT, elotuzumab target-mediated elimination rate from the peripheral compartment; $KINT_{Mono}$, effect of elotuzumab monotherapy on KINT; $KINT_{PomDex}$, effect of pomalidomide/dexamethasone administration on KINT; $KINT_{REF}$, typical internalization rate of the target from the peripheral compartment with lenalidomide/dexamethasone administration; KM, Michaelis–Menten constant of the target-mediated elimination from the central compartment; MCPROT, effect of serum M-protein; NA, not applicable; PPK, population pharmacokinetics; Q, inter-compartment clearance; Q_{REF}, typical inter-compartment clearance at reference values of covariates (WT = 75 kg); Q_{WT}, power coefficient of Q dependence on body weight; RMAX, baseline target concentration in the peripheral compartment; RSE, relative standard error; SD_{50}, elotuzumab concentration when standard deviation of the exponential error model is equal to $(SD_L + SD_H)/2$; SD_H, standard deviation of the exponential residual error model at high concentrations; SD_L, standard deviation of the exponential residual error model at low concentrations; $SD_{phase1,2}$, phase 1–2 study effect on the magnitude of the residual error; VC, volume of the central compartment; $VC_{B2MICG >0.35}$, B2MICG >0.35 mg/dL effect on VC; VC_{RACE}, Asian race effect on VC; VC_{REF}, typical volume of the central compartment at reference values of covariates (WT = 75 kg, male, non-Asian, B2MICG <0.35 mg/dL); VC_{SEX}, female sex effect on VC; VC_{WT}, power coefficient of VC dependence on body weight; VMAX, maximum target-mediated elimination rate from the central compartment; $VMAX_{MCPROT}$, effect of serum M-protein on VMAX; $VMAX_{REF}$, typical maximum target-mediated elimination rate from the central compartment at reference values of covariates (MCPROT = 0 g/dL); VP, volume of the peripheral compartment; VP_{REF}, typical volume of the peripheral compartment at reference values of covariates (WT = 75 kg); VP_{WT}, power coefficient of VP dependence on body weight; WT, weight

Supplementary Table S3 Parameter estimates for the full exposure–response model of efficacy (progression-free survival)

Predictor (Comparator: Reference)	Coefficient	SE	RSE (%)	Hazard ratio (95% CI)
Daily C_{avg} (ug/mL)	–0.00171	0.000696	40.82	0.9983 (0.9969–0.9997)
Baseline LDH (time of LDH_{ULN})[a]	0.8743	0.2953	33.77	2.397 (1.344–4.276)
Chromosomal abnormality t(4,14) (Yes:no)	0.6255	0.3831	61.24	1.869 (0.8822 - 3.96)
Baseline beta-2 microglobulin (mg/dL)[a]	0.8792	0.3032	34.49	2.409 (1.33–4.365)
Refractory status to lenalidomide (Yes:no)	0.3935	0.3978	101.1	1.482 (0.6797–3.232)
Refractory status to protease inhibitors (Yes:no)	0.03486	0.3454	990.9	1.035 (0.5262–2.038)
Prior stem cell transplantation (Yes:no)	0.2514	0.2748	109.3	1.286 (0.7504–2.203)
Time from disease diagnosis (≥Median:<Median)	–0.3891	0.2698	69.33	0.6777 (0.3994–1.15)
Baseline kappa light chain (mg/dL)	0.01148	0.07618	663.8	1.012 (0.8712–1.174)
Baseline lambda light chain (mg/dL)	–0.0085	0.07858	924	0.9915 (0.85–1.157)
Baseline urine M-protein (mg/day)	–0.02271	0.02856	125.7	0.9775 (0.9243–1.034)

[a]LDH/LDH_{ULN} and baseline beta-2 microglobulin have been log-transformed

C_{avg}, average concentration; CI, confidence interval; LDH, lactate dehydrogenase; RSE, relative standard error; SE, standard error; ULN, upper limit of normal

Supplementary Table S4 Parameter estimates for the full exposure–response model of safety (grade 3+ adverse events)

Predictor (Comparator: reference)	Coefficient	SE	RSE (%)	Hazard ratio (95% CI)
Daily C_{avg} (ug/mL)	–0.001893	0.000862	45.54	0.9981 (0.9964–0.9998)
Baseline beta-2 microglobulin (mg/dL)	0.8445	0.2974	35.22	2.327 (1.299–4.168)
ECOG status (1:0)	0.02065	0.252	1220.18	1.021 (0.6229–1.673)
ECOG status (2:0)	0.4304	0.3865	89.79	1.538 (0.721–3.28)

Hazard ratio coefficient represents the hazard ratio for one unit of change in the predictor variable. Baseline beta-2 microglobulin has been log-7ytransformed

C_{avg}, average concentration; CI, confidence interval; ECOG, Eastern Cooperative Oncology Group; RSE, relative standard error; SE, standard error

Supplementary Table S5 Parameter estimates for the full exposure–response model of efficacy without Pd only patients (sensitivity analysis; progression-free survival)

Predictor (Comparator: Reference)	Coefficient	SE	RSE (%)	Hazard ratio (95% CI)
Daily C_{avg} (ug/mL)	-0.001537	0.001248	81.2	0.9985 (0.996 - 1.001)
Baseline LDH (time of LDH_{ULN})[a]	2.197	0.6653	30.28	8.996 (2.442 - 33.14)
Chromosomal abnormality t(4,14) (Yes:no)	0.6291	0.7047	112	1.876 (0.4714 - 7.466)
Baseline beta-2 microglobulin (mg/dL)[a]	1.209	0.5496	45.46	3.351 (1.141 - 9.84)
Refractory status to lenalidomide (Yes:no)	0.9316	0.8254	88.6	2.539 (0.5035 - 12.8)
Refractory status to protease inhibitors (Yes:no)	-0.4863	0.5588	114.9	0.6149 (0.2057 - 1.839)
Prior stem cell transplantation (Yes:no)	0.9453	0.4753	50.28	2.573 (1.014 - 6.533)
Time from disease diagnosis (≥Median:<Median)	-0.7777	0.4771	61.35	0.4594 (0.1804 - 1.17)
Baseline kappa light chain (mg/dL)	0.1679	0.129	76.84	1.183 (0.9185 - 1.523)
Baseline lambda light chain (mg/dL)	0.2497	0.1482	59.36	1.284 (0.96 - 1.716)
Baseline urine M-protein (mg/day)	-0.1478	0.053	35.87	0.8626 (0.7775 - 0.9571)

[a]LDH/LDH_{ULN} and baseline beta-2 microglobulin have been log-transformed

C_{avg}, average concentration; CI, confidence interval; LDH, lactate dehydrogenase; RSE, relative standard error; SE, standard error; ULN, upper limit of normal

Supplementary Table S6 Parameter estimates for the full exposure–response model of safety without Pd only patients (sensitivity analysis; grade 3+ adverse events)

Predictor (Comparator: reference)	Coefficient	SE	RSE (%)	Hazard ratio (95% CI)
Daily C_{avg} (ug/mL)	-0.002425	0.001797	0.1802	0.9976 (0.9941 - 1.001)
Baseline beta-2 microglobulin (mg/dL)	0.8354	0.4046	17.55	2.306 (1.043 - 5.096)
ECOG status (1:0)	-0.03428	0.3518	36.41	0.9663 (0.4849 - 1.926)
ECOG status (2:0)	0.4412	0.6664	42.87	1.555 (0.4211 - 5.739)

Hazard ratio coefficient represents the hazard ratio for one unit of change in the predictor variable. Baseline beta-2 microglobulin has been log-7ytransformed

C_{avg}, average concentration; CI, confidence interval; ECOG, Eastern Cooperative Oncology Group; RSE, relative standard error; SE, standard error

Supplementary Figure S1 Graphic representation and equations of the population pharmacokinetic model

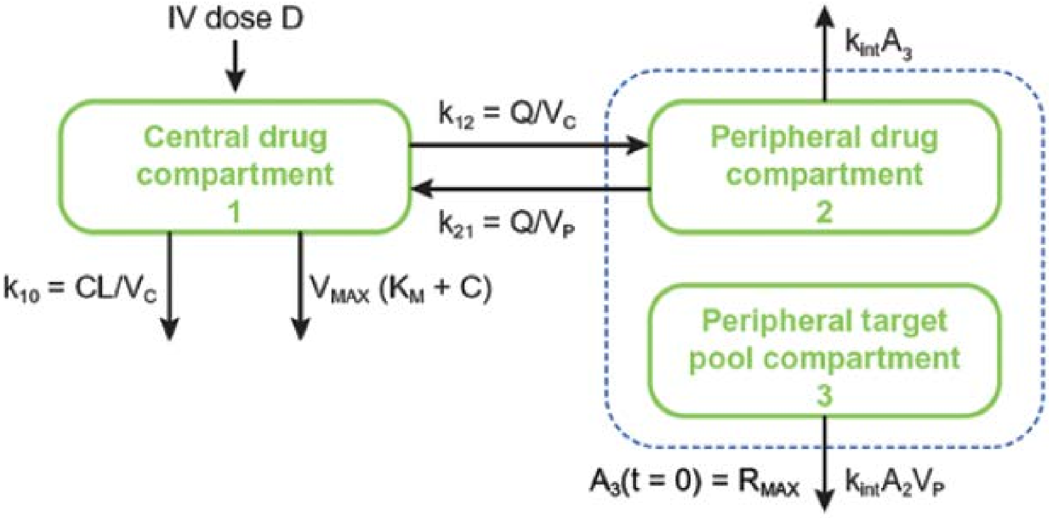

$$\frac{dA_1}{dt} = In(t) - \frac{CL}{V_C} A_1 - \frac{Q}{V_C} A_1 + \frac{Q}{V_P} A_2 - \frac{V_{MAX}\, A_1}{K_M + A_1/V_C};\ A_1(t = 0) = 0;$$

$$\frac{dA_2}{dt} = \frac{Q}{V_C} A_1 - \frac{Q}{V_P} A_2 - k_{int} A_2 A_3;\ A_2(t = 0) = 0;$$

$$\frac{dA_3}{dt} = - k_{int} \frac{A_2}{V_P} A_3;\ A_3(t = 0) = R_{MAX}$$

The two-compartment model was parameterized as follows: nonspecific (linear) clearance (CL), volume of distribution of central compartment (V_C), intercompartmental clearance (Q), volume of distribution of peripheral compartment (V_P), maximum rate of Michaelis–Menten elimination (V_{MAX}), Michaelis–Menten constant (KM), initial target SLAMF7 concentration in the peripheral compartment (R_{MAX}), second-order elimination rate constant of the drug-target complex from the peripheral compartment (k_{int}), infusion rate In(t).

Supplementary Figure S2 Impact of covariates on PK parameters of elotuzumab in the full population PK model

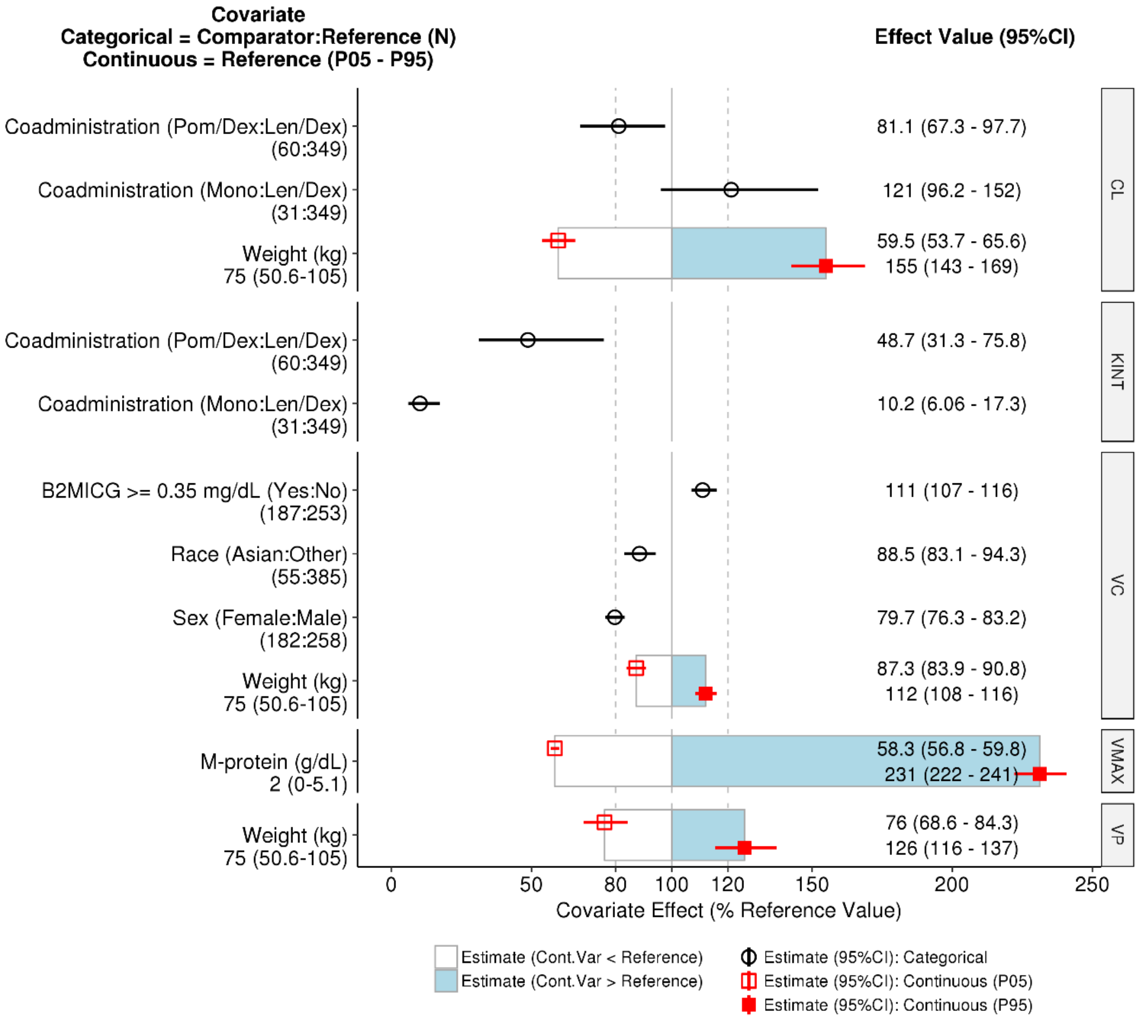

Continuous covariate effects (95% CI) at the 5th/95th percentiles of the covariate are represented by the end of horizontal boxes (horizontal lines). Open/shaded area of boxes represents the range of covariate effects from the median to the 5th/95th percentile of the covariate. Categorical covariate effects (95% CI) are represented by open circles (horizontal lines). Parameter estimate in reference subject is considered as 100% (solid vertical line) and the dashed vertical lines represent 80–120% of this value

B2MICG, serum beta-2 microglobulin; CI, confidence interval; cont. var, continuously varying; CL, clearance; dex, dexamethasone; KINT, target-mediated elimination rate from the peripheral compartment; len, lenalidomide; mono, monotherapy; P05–P95, $5^{th}/95^{th}$ percentiles; pom, pomalidomide; VC, volume distribution of the central compartment; VMAX, the maximum rate of Michaelis–Menten elimination; VP, volume of distribution of the peripheral compartment

Supplementary Figure S3 Model evaluation for the final exposure–response model of efficacy (progression-free survival)

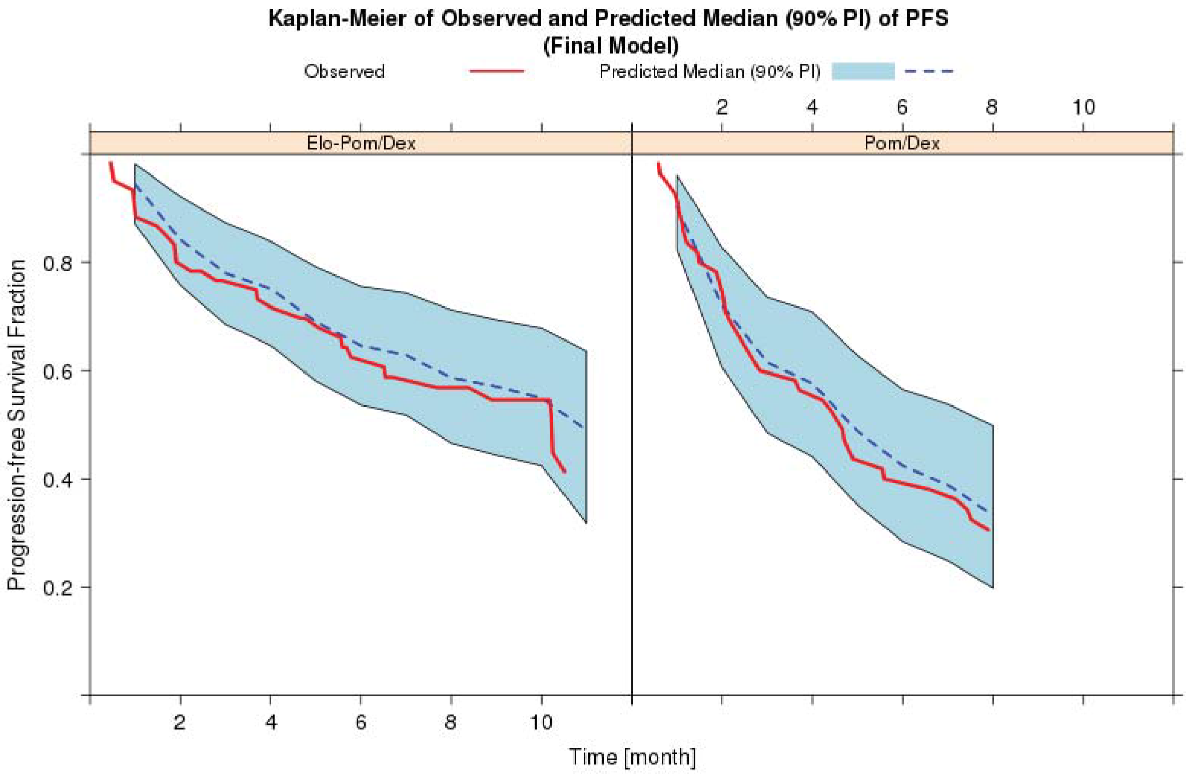

Dex, dexamethasone; elo, elotuzumab; PFS, progression-free survival; PI, predicted interval, pom, pomalidomide

Supplementary Figure S4 Model evaluation for the final exposure–response model of safety (grade 3+ adverse events)

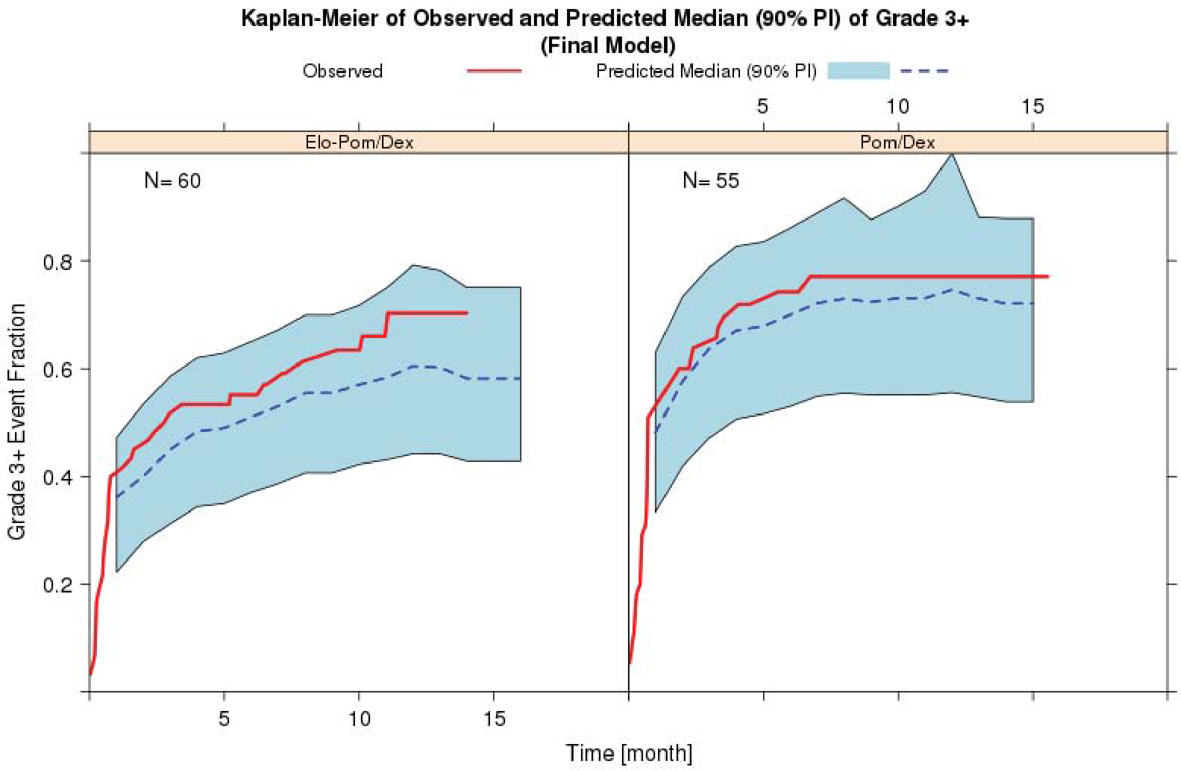

Dex, dexamethasone; elo, elotuzumab; PI, predicted interval; pom, pomalidomide
